# Spectroscopic ellipsometric study datasets of the fluorinated polymers: Bifunctional urethane methacrylate perfluoropolyether (PFPE) and polyvinylidene fluoride (PVDF)

**DOI:** 10.1016/j.dib.2021.107461

**Published:** 2021-10-09

**Authors:** Joseph Gibbons, Samuel B.H. Patterson, Adilet Zhakeyev, Filipe Vilela, Jose Marques-Hueso

**Affiliations:** a Institute of Sensors, Signals and Systems, School of Engineering and Physical Sciences, Heriot-Watt University, Edinburgh, Scotland EH14 4AS, UK; b Institute of Chemical Sciences, School of Engineering and Physical Sciences, Heriot-Watt University, Edinburgh, Scotland EH14 4AS, UK

**Keywords:** PFPE, PVDF, Refractive index, Transmission intensity, Optical absorbance, Spectroscopic ellipsometry, Polymer optical properties

## Abstract

The datasets in this work contain the experimentally measured (real) refractive indices, optical transmission intensity, and optical absorption spectra of bifunctional urethane methacrylate perfluoropolyether (PFPE; Fluorolink® MD700) substrate of (0.98 ± 0.13) mm thickness and polyvinylidene fluoride (PVDF; Kynar® 705) thin-film of (4.47 ± 0.29) µm thickness over a spectral range from 300 nm to 1000 nm, as measured *via* variable angle spectroscopic ellipsometry. The refractive indices data were determined by employing a single Cauchy optical constants function based layer using a Levenberg-Marquardt multi-iterative regression algorithm for all model minimizations. The mean-squared error (MSE) was used as the maximum likelihood estimator, with a convergence of the Levenberg-Marquardt algorithm reached when successive iterations were unable to improve the MSE. The resulting best-fit parameter values were evaluated for sensitivity (expressed as a confidence limit), and possible correlations. Furthermore, the experimentally measured optical transmission intensity and determined optical absorption of PFPE and PVDF, over a spectral range from 300 nm to 1000 nm, is also presented, as measured *via* ellipsometry and corrected using Fresnel equations to accommodate surface interference. Given the high transmission of (88.4 ± 0.5)% for PFPE and (95.6 ± 0.6) % for PVDF found, and the low refractive index 1.27 (λ = 589.3 nm) found for PFPE; it is thought that these datasets may be useful for optical applications, such as for photo-curable synthesis processes, or being used as a host-matrix material for photoluminescent compounds.

**Specifications Table**

**Table:** 

Subject	Polymers and Plastics
Specific subject area	Ellipsometric properties of a ultraviolet (UV) cross-linked PFPE substrate and a spin-coated PVDF thin-film fluoropolymer samples
Type of data	Figures
How data were acquired	The ellipsometric properties of the PFPE and PVDF samples were analyzed using a variable-angle spectroscopic ellipsometer (J.A. Woollam Base VASE)
Data format	Raw analyzed
Parameters for data collection	For the refractive index measurements, the samples were analyzed over a spectral range of 300 nm to 1000 nm with a resolution of 10 nm, at 60 revs/meas, over angles of incident angles 65°, 70°, and 75°. For the optical transmission measurements, the samples were analyzed over a spectral range of 300 nm to 1000 nm with a resolution of 1 nm, at 60 revs/meas, with an angle of incident of 0°.
Description of data collection	A spectroscopic scan was conducted with the change in polarization state of light measured. The refractive indices are determined from a single isotropic Cauchy optical constants function based layer, using an iterative Levenberg-Marquardt fitting algorithm. The optical absorbance of the samples were determined by measuring the transmission intensity. The sample was inserted into the beam path with an angle of incidence of 0°, the probe beam was intercepted by the detector with the polarization state transmitted by the substrate measured.
Data source location	Heriot-Watt University, Edinburgh, Scotland, EH14 4AS
Data accessibility	Repository name: Data for: spectroscopic ellipsometric study of the fluorinated polymers bifunctional urethane methacrylate perfluoropolyether (PFPE) and polyvinylidene fluoride (PVDF) Data identification number: 10.17632/bwtzc9rzb9.2 Direct URL to data: https://data.mendeley.com/datasets/bwtzc9rzb9/2

**Value of the Data**

• The fluoropolymers PFPE and PVDF exhibit properties which make them suitable candidates for widespread and demanding applications due to their excellent chemical and thermal resistance, with extreme transparency. However, the accurate determination of their refractive index dispersion is not explored in the literature.
• The experimentally measured ellipsometric data provides useful insight into the optical properties of PFPE and PVDF and will benefit research fields that are extensively investigating applications, for example, anti-reflective, easy to clean, water and oil repellent coatings [1], low-refractive-index cladding for optical fibres [2], polymeric waveguides with low optical losses [3], and energy harvesting piezoelectric devices [4].
• The measured data of the specially prepared PFPE and PVDF presents useful information for the various applications it is used, and provides fundamental aid for optical and optoelectronic device design. Furthermore, it is possible to modify the synthesis protocol deployed here and thus, fine-tune the structure of the product to tailor their final properties to achieve specific optical performance.

## 1 Data Description

This Data in Brief article provides figures, and their respective datasets, from the ellipsometry study of the fluoropolymers PFPE and PVDF. Two types of datasets are provided within the repository as contained within a subfolder, as categorized by material type complete with their respective dataset description text files.

### 1.1 Ellipsometric indices of refraction

**Fig. 1:**
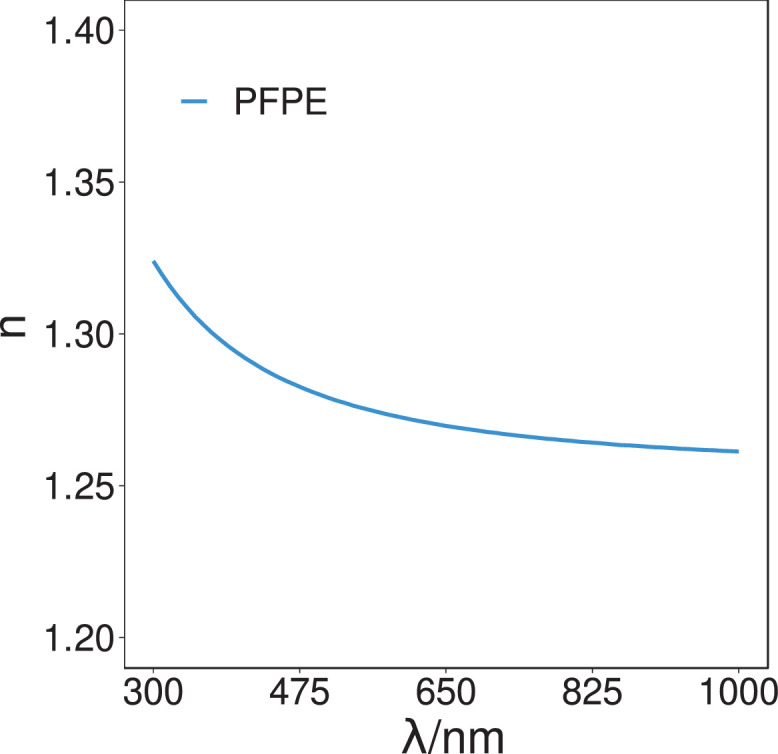
The ellipsometric (real) indices of refraction (n) values of the PFPE sample over the spectral range of 300 nm–1000 nm. Data derived from the Cauchy optical model using the Levenberg-Marquardt fitting algorithm. The refractive index was found to be 1.27 (λ = 589.3 nm).

**Fig. 2:**
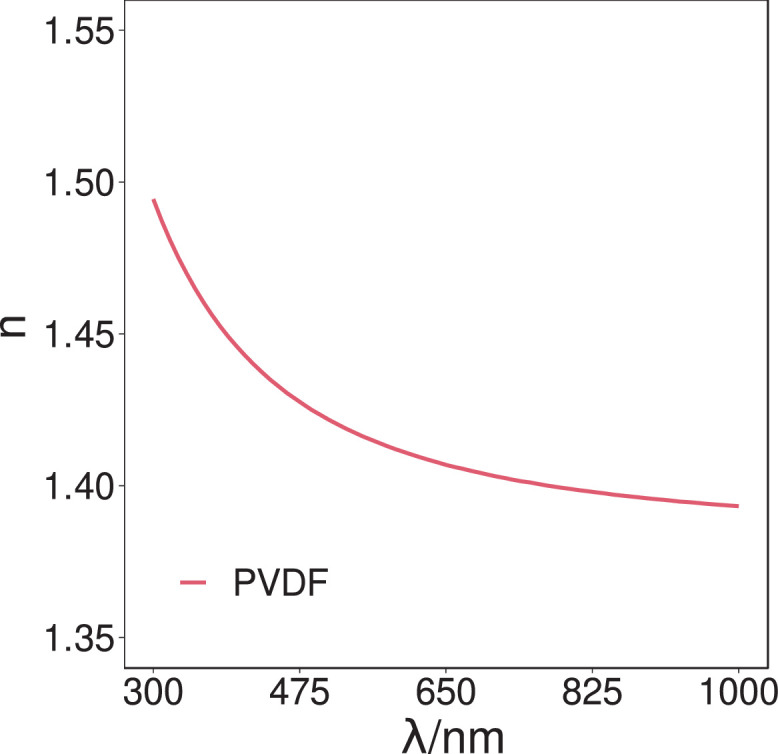
The ellipsometric (real) indices of refraction (n) values of the PVDF sample over the spectral range of 300 nm–1000 nm. Data derived from the Cauchy optical model using the Levenberg-Marquardt fitting algorithm. The refractive index was found to be 1.41 (λ = 589.3 nm).

Fig. 1 (spectroscopic-scan-PFPE-refractive-indices-cauchy-V1.csv) and Fig. 2 (spectroscopic-scan-PVDF-refractive-indices-cauchy-V1.csv) present the experimentally measured (real) indices of refraction (n) of the PFPE substrate and PVDF thin-film samples, as determined from the ellipsometric study, over the spectral range 300 nm–1000 nm, experimentally measured at a resolution of 10 nm. The data was modeled using a single isotropic Cauchy optical constants function based layer using incidence angles of 65°, 70°, and 75°.

### 1.2 Transmissivity intensity and optical absorbance

**Fig. 3:**
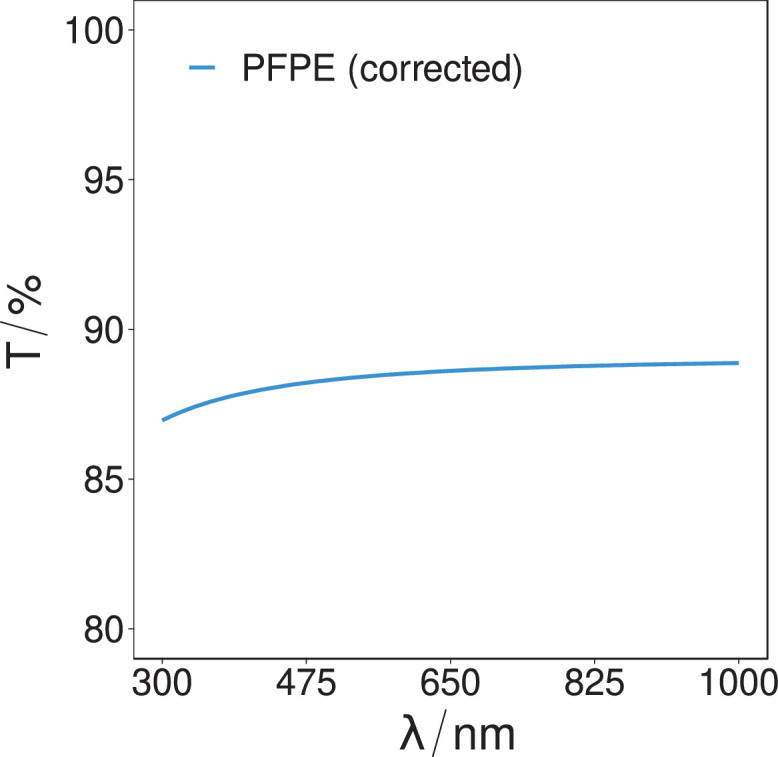
The corrected transmissivity intensity of the PFPE sample over the spectral range of 300 nm to 1000 nm. The mean transmissivity over the spectrum is determined to be (88.4 ± 0.5)%.

**Fig. 4:**
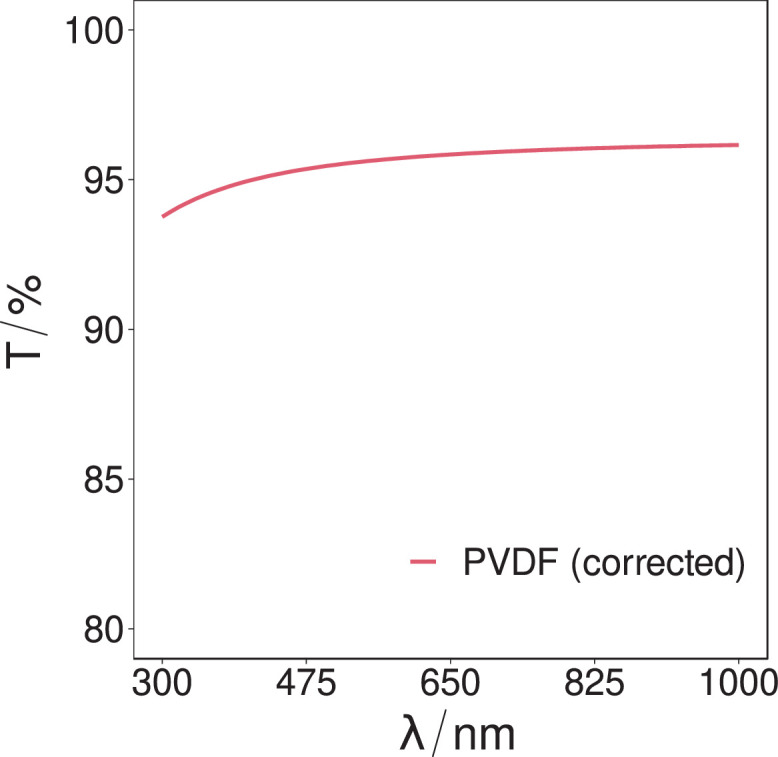
The corrected transmissivity intensity of the PVDF sample over the spectral range of 300 nm to 1000 nm. The mean transmissivity over the spectrum is determined to be (95.6 ± 0.6)%.

Fig. 3 (spectroscopic-scan-PFPE-transmission-absorption-V1.csv) and Fig. 4 (spectroscopic-scan-PVDF-transmission-absorption-V1.csv) present the experimentally measured, and subsequently corrected using the Fresnel equations, optical transmissivity spectra of the PFPE substrate and PVDF thin-film samples, as determined from the ellipsometric study over the spectra range of 300 nm–1000 nm measured at a resolution of 1 nm.

**Fig. 5:**
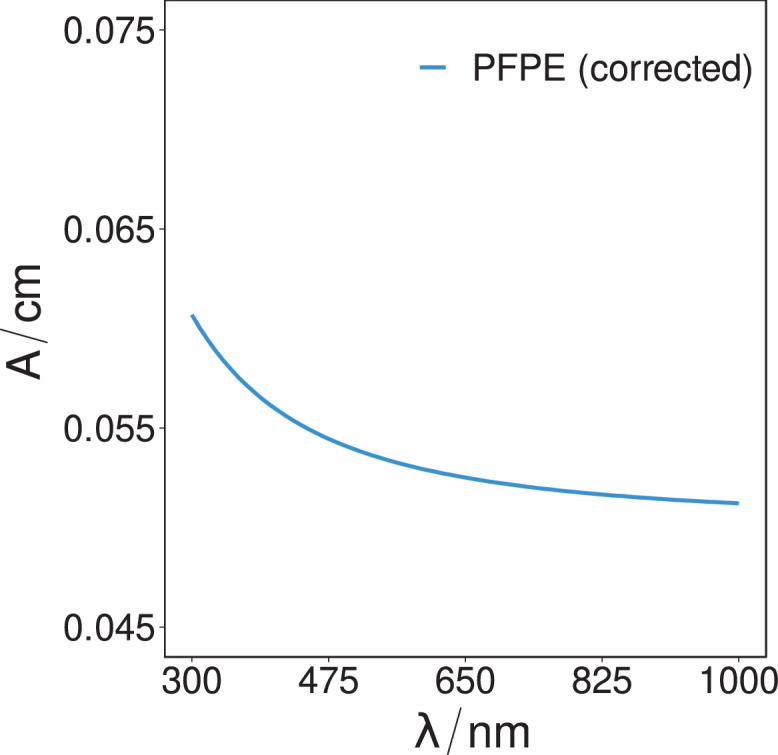
The determined optical absorbance coefficients of the PFPE sample over the spectral range of 300 nm to 1000 nm, as calculated from the corrected transmissivity intensity.

**Fig. 6:**
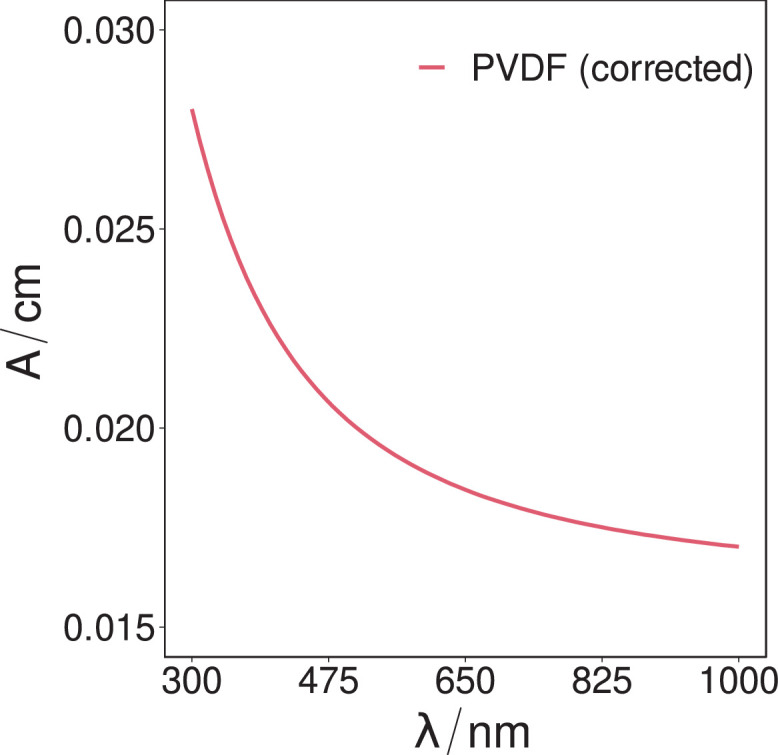
The determined optical absorbance coefficients of the PVDF sample over the spectral range of 300 nm to 1000 nm, as calculated from the corrected transmissivity intensity.

The determined optical absorption coefficient of the PFPE substrate and PVDF thin-film samples, as calculated from the transmission intensity data over the same spectral range of 300 nm–1000 nm, is presented in Fig. 5 (spectroscopic-scan-PFPE-transmission-absorption-V1.csv) and Fig. 6 (spectroscopic-scan-PVDF-transmission-absorption-V1.csv).

## 2 Experimental Design, Materials and Methods

### 2.1 PFPE sample preparation

Bifunctional urethane methacrylate perfluoropolyether (PFPE; Fluorolink® MD700) was processed to form a bulk substrate. A stock solution of 1 mL acetone and 18.75 mg of photoinitiator diphenyl (2,4,6 - trimethylbenzoyl) phosphine oxide (TPO) was prepared, 3 mL of PFPE was added to this to achieve a solution with a final concentration of 6.25 mg/mL, which was mixed *via* magnetic stirring. After mixing, entrapped air bubbles were removed by using an ultrasonic bath at a temperature of 20 °C. The solution was then poured into a stainless steel mold cast and cured *via* an ultraviolet crosslinker (UVP CL-1000) by being exposed to 254 nm for 15 min at an energy irradiance level of 120 mJ/cm^2^. The prepared PFPE sample dimensions measured (22.78 ± 0.04) mm × (23.16 ± 0.09) mm with a thickness of (0.98 ± 0.13) mm.

### 2.2 PVDF sample preparation

Polyvinylidene fluoride (PVDF; Kynar® 705) was dissolved in *N,N*-dimethylformamide (DMF) at a concentration of 20 wt% and heated to a temperature of 60 °C to accelerate the solubility process, and stirred *via* a magnetic stirrer for 60 min. Following this, the mix was spin coated onto a circular 1” diameter glass wafer at 1250 rpm for 15 s. After spin coating, to ensure complete removal of the DMF solvent, the sample was simultaneously dried and annealed at 60 °C for 120 min on a hot-plate. The prepared PVDF thin-film had a final thickness of (4.47 ± 0.29) µm.

### 2.3 Instrument performance assessment and calibration

Prior to conducting the measurements, the hardware signal of the instrument was checked to examine the raw signal that the system was acquiring, observed with the monochromator set to 5000 Å, and the number of analyser revolutions set to 10 revs/meas. The signal displayed an 18-period sinusoidal waveform, enveloped by a 2-period sinusoidal waveform, with experimental and fitted curves showing good correlation, demonstrating a normal – and therefore acceptable – signal.

The alignment procedure was then conducted to ensure that the standard calibration sample surface was perpendicular to the probe beam. A standard 76.2 mm diameter silicon (Si) wafer with a thermally grown fused silica (SiO_2_) layer approximately 25 nm thick sample was used for the alignment and calibration procedures. The angle of incidence for each calibration procedure was aligned to *X* = (0.0 ± 0.1)° and *Y* = (0.0 ± 0.1)°, correct to within the accuracy of the goniometers = (0.000 ± 0.005)°, and with a gain of < 10 achieved. The Z-translation alignment procedure was then conducted, with values of 500 nm and 70° selected, to ensure that the sample surface was placed on the center of rotation of the sample stage, and that the probe beam would enter the center of the detector unit at all angles. The *Z*-axis micrometre was adjusted until the maximum signal was found.

Following this, a regression calibration procedure was conducted using the standard SiO_2_/Si calibration sample, to align the transmitting axes of the polarizer and the analyser relative to the sample plane of incidence. The input polarizer was scanned over a range of positions with the polarizer optical axis on either side of the plane of incidence. The degree span was set to 100° (nominally, −50° to 50°), that is, the range of the polarizer setting in which the data was acquired. At each of the polarizer settings, the Fourier coefficient data were then fit to determine the calibration constants. The calibration yielded a perfect fit and identical calibration parameters, indicating the model was accurate and the ellipsometer was functioning ideally.

The alignment procedure was then repeated for the PFPE substrate and PVDF thin-film samples, accordingly. As before, the angle of incidence was aligned to *X* = (0.0 ± 0.1)° and *Y* = (0.0 ± 0.1)°, correct to within the accuracy of the goniometers = (0.000 ± 0.005)°, and with a gain of < 10 achieved. The *Z*-translation alignment procedure was again repeated for the samples, with the *Z*-axis micrometre adjusted until the maximum signal was found.

### 2.4 Spectroscopic ellipsometry and analysis

The experimental spectroscopic ellipsometric measurements were conducted using a varied-angle spectroscopic ellipsometer with light incident angles of 65° to 75°, at 5° increments, selected as acceptable as the delta data for these three angles of incidence where close to ∆ = 0° or ∆ = 180° [5], [6], [7]. The ellipsometry data, taken from the sample substrate, was fit, in a non-linear regression sense, to a single isotropic Cauchy optical constants function based layer to determine the (real) indices of refraction. The Cauchy function describes the dispersion over part of the spectral range, and fits the optical constants using the iterative Levenberg-Marquardt fitting algorithm [8,9]. The methodology follows that of the ones presented previously [10,11].

Each sample measurement was repeated at each incident angle once (*n* = 1). The (real) indices of refraction (n) are represented by a slowly varying function of wavelength (λ) [5,12]. Simulated standard deviations were calculated to approximate the relative noise levels of data acquired with different ψ and ∆ combinations, and were derived by assuming the measured normalized Fourier coefficients α and β are constant. The mean-squared error (MSE) was used as the maximum likelihood estimator, defined to represent the quality of the match between data calculated from the model and the experimental data, and was found to be 5.757 for PFPE and 6.896 for PVDF.

### 2.5 Optical transmission intensity and absorption coefficient

The optical absorbance of the PFPE substrate and the PVDF thin-film was determined by first measuring the optical transmission intensity using a varied-angle spectroscopic ellipsometer. The transmissivity intensity measurements acquired with the instrument are performed using a ‘straight through no’ sample intensity scan (i.e., full probe beam) as the baseline. The instrument, therefore, calculates absolute intensity transmission values from 0 to 1. The instrument acquires polarized transmissivity intensity data, that is, the incident beam has a single, known polarization state. The ellipsometer detects the intensities for the polarization component of the transmitted beam.

The detector remained fixed at 180° (i.e., normal incidence) while the measurements were performed, and the resulting data marked as ellipsometric transmission intensity data. The ellipsometer uses the single beam technique, and thus required a baseline scan and main data scan for the measurement of the transmission intensity. As the single beam technique is susceptible to errors due to fluctuations or drift of the light source intensity, the baseline scan and main scans were acquired in close succession. Normal incidence transmission measurements were conducted as they are the easiest measurement to perform accurately. The sample was inserted into the beam path after the baseline measurement, whereby the probe beam does not deviate away from the detector, thus avoiding systematic errors in the resulting transmission intensity measurement (which would otherwise be caused by the probe beam potentially being offset whilst passing through the sample). For the transmission scan, p-polarized data type (pT: trans p-pol) was selected and the polarizer moved to 0°, thus, the electric field of the incident beam was linearly polarized.

Dynamic averaging was enabled during the measurements, which determined the number of analyzer cycles by averaging the data until the standard deviation was acceptable. This was done to ensure an experimental data set was yielded with an approximately constant standard deviation where possible. The maximum number of analyser cycles per measurement was set to 60 revs/meas. The monochromator slit width used for the data acquisition was 1900 µm, with slit auto-averaging permitted (min = 100 µm) to allow the monochromator to adjust to ensure the intensity was kept within the optimum range for the detector.

With the raw transmission intensity measurements conducted, Fresnel equations were used to describe the transmission of electromagnetic waves at an interface (i.e., the surface of the substrate) at each measured wavelength. The equations provide the transmission coefficients for waves parallel and perpendicular to the plane of incidence, as defined by Jackson [13,14]:

$$t_{par}=\frac{2sin\left(\theta_{t}\right)cos\left(\theta_{i}\right)}{sin\left(\theta_{i}+\theta_{t}\right)cos\left(\theta_{i}-\theta_{t}\right)}$$

$$t_{per}=\frac{2sin\left(\theta_{t}\right)cos\left(\theta_{i}\right)}{sin\left(\theta_{i}+\theta_{t}\right)}$$

where $\theta_{i}$ is the angle of incidence, $\theta_{t}$ is the angle of transmission, $t_{par}$ is the parallel transmission coefficient, and $t_{per}$ is the perpendicular transmission coefficient.

As these coefficients are fractional amplitudes; they must be squared to get fractional intensities. With these values known for a given scenario, applying the conservation of energy leads to the following relationship [13,14]:

$$r^{2}+t^{2}\frac{n_{1}cos\left(\theta_{t}\right)}{n_{2}cos\left(\theta_{t}\right)}=1$$

where $n_{1}$ is the refractive index of the medium that the beam travels (i.e., air), and $n_{2}$ is the refractive index incident on the medium (i.e., the substrate). Note: this applies to both the parallel and perpendicular cases.

Once the transmission spectra of each substrate were determined, the substrates optical absorbance per cm (base 10) was then determined by [15]:

$$A/cm=2-log_{10}\left(T\right)$$

where *T* is the transmission, and *A/cm* is the optical absorbance per centimetre (base 10).

Ellipsometric transmission-based measurements require that the thickness of the sample is optimized for the dynamic range of the technique so that the transmittance of the sample is > 1%, otherwise, the accuracy of the measurement is degraded [5]. The inherent sensitivity of spectral transmission and absorbance measurements is affected by the optical path length of the sample and the decrease in transmission that occurs.

## Ethical Statement

As per the duties under the publishing ethics standard the authors declare that, to the best of our knowledge, this submission reports original research that presents an accurate account of the work performed as well as an objective discussion of its significance. All underlying data is represented accurately in the paper. Furthermore, the authors are unaware of any fraudulent or knowingly inaccurate statements.

## CRediT authorship contribution statement

**Joseph Gibbons:** Methodology, Formal analysis, Investigation, Writing – original draft, Visualization. **Samuel B.H. Patterson:** Methodology, Writing – review & editing. **Adilet Zhakeyev:** Methodology, Writing – review & editing. **Filipe Vilela:** Methodology, Resources, Writing – review & editing, Funding acquisition. **Jose Marques-Hueso:** Conceptualization, Writing – review & editing, Supervision, Funding acquisition.

## Declaration of Competing Interest

The authors declare that they have no known competing financial interests or personal relationships which have, or could be perceived to have, influenced the work reported in this article.
